# Evaluation of health resource utilization efficiency in community health centers of Jiangsu Province, China

**DOI:** 10.1186/s12960-018-0275-y

**Published:** 2018-02-20

**Authors:** Xinglong Xu, Lulin Zhou, Henry Asante Antwi, Xi Chen

**Affiliations:** School of Management, Jiangsu University, No.301 Xuefu road, Zhenjiang, 212013 Jiangsu Province People’s Republic of China

**Keywords:** Efficiency, Health resources, Urban communities, China’s Jiangsu Province

## Abstract

**Background:**

While the demand for health services keep escalating at the grass roots or rural areas of China, a substantial portion of healthcare resources remain stagnant in the more developed cities and this has entrenched health inequity in many parts of China. At its conception, China’s Deepen Medical Reform started in 2012 was intended to flush out possible disparities and promote a more equitable and efficient distribution of healthcare resources. Nearly half a decade of this reform, there are uncertainties as to whether the attainment of the objectives of the reform is in sight.

**Methods:**

Using a hybrid of panel data analysis and an augmented data envelopment analysis (DEA), we model human resources, material, finance to determine their technical and scale efficiency to comprehensively evaluate the transverse and longitudinal allocation efficiency of community health resources in Jiangsu Province.

**Results:**

We observed that the Deepen Medical Reform in China has led to an increase concern to ensure efficient allocation of community health resources by health policy makers in the province. This has led to greater efficiency in health resource allocation in Jiangsu in general but serious regional or municipal disparities still exist. Using the DEA model, we note that the output from the Community Health Centers does not commensurate with the substantial resources (human resources, materials, and financial) invested in them. We further observe that the case is worst in less-developed Northern parts of Jiangsu Province.

**Conclusions:**

The government of Jiangsu Province could improve the efficiency of health resource allocation by improving the community health service system, rationalizing the allocation of health personnel, optimizing the allocation of material resources, and enhancing the level of health of financial resource allocation.

## Background

The global effort to eradicate extreme inequity in socioeconomic and health wellbeing of all mankind especially those living on the fringes of society is evident in the numerous social intervention policies that have been rolled out by the United Nations and other international organizations [1]. These initiatives include the United Nations Millennium Development Goals and the 2011 Rio Summit on the Social Determinants of Health. In China, eradication of health inequity remains an adventure in transition [2]. The theme of the “Healthy China 2020” which is a program launched by the government to provide universal healthcare access and treatment for all in China and reduce health inequities by the year 2020 exemplifies this notion. The preamble to the reform policy calls for an augmented central and local government effort to improve equity, restructure public health functions, and rejuvenate existing national policies on health inequities [3, 4]. Predictably, China achieved universal health insurance coverage in 2011, representing the largest expansion of insurance coverage in human history. This success was attained because the government’s renewed commitment for better healthcare reform enjoyed strong public support across China [2]. In this connection, both the central government provided heavy subsidies to support industries involved in producing healthcare products, and greater support for enrolment into new social health insurance programs were initiated [2]. The idea to decentralize decision making in healthcare to local governments contributed significantly to pragmatic implementation strategy for health equality.

In 2015, a post-reform audit was conducted and the report published in the China National Health Equality Survey. The results showed that even though improvement had been recorded in the general population’s health status, inequity in access to healthcare had not been completely eradicated in many parts of China [5]. The report specifically indicated that some critical diseases are not covered under the social health insurance system and this is a source of health inequity. Moreover, financial, human, and technological resources to deliver quality healthcare were concentrated in relatively richer provinces such as Jiangsu, Guangdong, Shanghai, and Beijing. This is the exact opposite of what happens in poorer western provinces such as Tibet, Gansu, Xinjiang, Yunnan, and Qinghai. For this reason, there is the need to continuously search for a sustainable healthcare reform plan for China. In another study, Zhou et al. [5] argue that the structure of the health system and the mechanism for allocating health resources play a vital role in health-promoting health equity. It is their considered opinion that these are highly misaligned and contribute to health inequity in China [6].

The basic structure of healthcare organization in China is also a source of inequity in access to healthcare. China organizes its healthcare system on a three-tier system in both the urban and rural areas [7] In the rural areas, village clinics and township health centers provide most of the primary healthcare services while county hospitals provide specialty medical services. In the urban areas, however, the community health centers and district hospitals provide primary healthcare. The municipal and provincial hospitals in the urban areas provide tertiary medical services to both urban and rural people [8].

Over a period of two decades, this basic structure of healthcare delivery has expanded very rapidly in terms of number of facilities and personnel but the management system has not changed significantly [5]. For example, at the end of 2015, there were nearly 1 006 000 health centers, including 915 000 village clinics, 18 800 general hospitals, 54 300 township health centers, 54 600 centers for disease control (CDCs) (which provide public health programmers addressing infectious diseases, health education, food security, environmental health, etc.), 30 000 maternal and child care centers, and 17 000 disease-specific treatment centers.

This is in addition to nearly 1.87 million village health workers and 5.3 million health workers in township and higher-level health centers (Ministry of Health, [9]). Despite these expansions, the 2015 China National Health Equality Survey (as reported by Dobbelaere and Mairesse [10]) indicates that infant and childhood mortality, life expectancy, maternal mortality, doctor to patient ratio, health insurance coverage, and other indicators of health equity are better in richer provinces such as Jiangsu, Shanghai, Guangdong, Zhejiang, and Shandong.

An examination of key themes in series of recent studies on China’s healthcare sector [2, 11] also attributes persistent imbalance to factors including the distributive injustice or mismatch between the allocation of health care resource and health service demand in most Chinese communities. In rural areas, besides the limitations due to financial resources, there is also shortage of qualified health workers. More than 75% of the medical officers working in village clinics are high school graduates with 6 months of training at a county or community hospitals. These are popularly called “barefoot doctors” because they have received very little medical training for their operations (Wang, 2003). Additionally, only 18.7% of township health workers are educated at medical university compared to 41% in cities (Ministry of Health, [12]). This is consistent with the observation of Guo [11] who notes that the demand for primary healthcare at the community level remains the largest source of healthcare demand, yet most of the resources are concentrated at the larger medical centers in major cities and towns thereby denying the largest proportion of those that need healthcare the ease of access [3]. While the rural-urban disparity in allocation of healthcare resources is widespread in China, the exacerbating trend in the Jiangsu Province has triggered a search for a more sustainable metric to ensure effective allocation of healthcare resources. Being the fifth most populous and the most densely populated province of the 22 provinces of the People’s Republic of China, Jiangsu has the second highest GDP of Chinese provinces after Guangdong. The Provincial Department of Health (2015) reports that hospital bed utilization rate tumbled by nearly 0.98 percentage points in 2005 to 48.9% in 2013, indicating a low efficiency of allocation of health resources [4]. These and many other challenges facing the healthcare sector in Jiangsu Province significantly undermines the World Health Organization proposed health resource allocation principles of equity and efficiency. Thus, the national and provincial search for a more systematic and sustainable approach to improving efficient allocation of health resources has led to the proposal and experimentation of different programs and projects with different levels of successes and challenges. It is the contention of Zhou et al. [5] that the hope that the deepened national medical reform initiated in 2012 will bring efficiency in community health resource allocation has become a mirage owing to the conflicting views regarding the extent to which healthcare resource allocation has been improved under the new policy.

These conflicts relate to differences in measurement metrics of efficiency as well as variables used in measuring them. Other differences have also emanated from the models used in evaluating efficiency utilization of resources requiring a more robust approach. In the extant literature, Farrell proposes a model for evaluating efficiency based on the concept of configuration efficiency, pure technical efficiency, and scale efficiency in the early stage [13]. Since then, several other models for evaluating efficiency have been proposed in different areas, such as energy [6], science and technology [7], and environment [8]. Li et al. [14] proposed the data envelopment analysis (DEA) model and has since been rapidly applied to medical and health sectors [10]. On the other hand, Ahn and Schmidt [3] were the earliest to use panel data in economic analysis when scholars began to consider time and cross-sectional influences on the efficiency evaluation [13]. To the best of our knowledge, there is little attempt to apply a hybrid of time series data and DEA data to evaluate resource allocation efficiency in the health sector. This study therefore uses the DEA method to analyze efficiency utilization of human, financial, and other resources invested in using panel data analysis. Emphasis is placed on technical and scale efficiency to evaluate allocation of community health resources in Jiangsu Province transversely and longitudinally.

In terms of public health or epidemiological contribution, this study is an ecological cross-sectional study. It is ecological because it seeks to study risk-modifying factors on health or other outcomes based on populations defined either geographically or temporally using statistical methods. The study is also cross-sectional because it analyzes data collected from a population at a specific point in time. The study evaluates the end results (outcomes) of the structure and processes of the health care system on the health and well-being of patients and populations. Thus, the intent of this research is to identify shortfalls in practice and to develop strategies to improve care.

## Methods

### Data collection

There are many community health centers (CHCs) in Jiangsu Province, but this study sampled data from 75 CHCs equally distributed in the three zones under review. Two reasons informed the selection of these specific 75 CHCs for the study. Firstly, they are designated as priority CHCs since 2013. For this reason, extended support in the form of technology, labour, capital, research, and other valuable resources has been invested into them by the provincial government to ensure high-quality and more efficient services. Secondly, the 75 CHCs were chosen because they fall within the research jurisdiction of the Institute of Medical Insurance and Hospital Management (IMIHM) of the Jiangsu University where this study was conducted. Research on other CHCs is conducted by other research centers in the province as part of measures to streamline data collection and research activities among designated organizations by the authorities.

Twenty-five (25) of the CHCs are in the most economically developed southern zone of Jiangsu Province (Nanjing, Zhenjiang, Suzhou, Wuxi, and Changzhou prefectures). Twenty-five (25) of them are also located in Yangzhou, Taizhou, and Nantong with relative economic development. These prefectures are located in the middle zone of Jiangsu Province. Finally, the remaining 25 CHCs were sampled from Xuzhou, Lianyungang, Suqian, Huaian, and Yancheng in the Northern zone of Jiangsu Province and are less economically active areas. The same number of CHCs was selected to provide a fair basis to make comparison of results. The Census and Statistics Department which keeps validated and administrative data for Jiangsu Province provided the data [15].

### Selection of input and output variables for DEA

The study used an input and output form of DEA; however, the efficiency of a decision making unit (DMU) or CHC is not dependent on the absolute value of the input variable but the outcome of the model. The guidelines for variable selection in a DEA by [14] were used to select 14 initial human resources, materials, financial, and service variables. This requires the selected variables to reflect the input and output situation of community, representativeness sample, elimination of multicolinearity among variables, consistent sample size-evaluation unit ratio, etc.

The 14 input variables initially selected for regression were number of centers, number of beds, number of health workers, number of general standardized training practitioners, financial investment, the cost per visit, proportion of CHC within an area of 3000 m^2^, and proportion of residents under grid management in communities CHC. The initial output variables selected included the number of discharged patients, cure rate, bed occupancy rate, average inpatient days, the daily number of outpatient and emergency visits of doctor, and the daily number of inpatient stays of doctor. The variable selection was done after searching related materials and consulting with health administrators, CHC managers, and experts.

Three methods were adopted to validate and assess trend and standardization of initial 14 variables using SPSS Version 19 before the final selection. Firstly, a cluster analysis was performed to help address the overlap between the capability of variables to explain the same portion of the outcomes and this helped to shortlist the essential and representative variables as recommended by [14], after which a correlation matrix was extracted to identify and eliminate multicolinearity among the variables. Thirdly, due to the susceptibility of DEA technique susceptible to a bad selection of variables (possibility of selecting input hat may not adequately explain variations in selected output), we formulated and conducted regression analysis to choose the inputs with high capability to explain a substantial amount of the variance of the outputs selected. A coefficient of determination value of 60% was used as benchmark to select the final list of input and output variables.

The regression model was complemented with the coefficient method to select final list of input and output variables based on the degree of dispersion from the shortlisted ones. Consistent with traditional production functions, labor and capital were treated as input variables in the production of health services in a Community Health Center. Labor was represented by (1) the number of doctors, (2) the number of nurses, (3) the number of pharmacists, and (4) the number of the other staff (medical staff and administrative workers). The number of beds was used as proxy for the CHCs’ capital stock (see [7, 14, 16]). On the other hand, the number of outpatient and inpatient cases was treated as outputs variables and were chosen for the 13 cities over an 8-year period for the final analysis [16–18].

Another important issue that was addressed was the case of controlling of weights of input and output variables. Li et al. (as cited in [14]) recognized the difficulty in seeking a common set of weights to determine relative efficiency. They recognized the legitimacy of the proposal that units might value inputs and outputs differently and therefore adopt different weights, and proposed that each unit should be allowed to adopt a set of weights which shows it in the most favorable light in comparison to the other units. To overcome this challenge, we employed a cross-efficiency approach proposed by Sexton et al. in 1986 and popularized by Doyle and Green’s [19] where each DMU peer appraises all other DMUs with its own factor weights.

### Data analysis

#### DEA model

The study utilizes the DEA model of C^2^R to evaluate the relative efficiency of decision making units (DMUs). This has been proven suitable for evaluating efficiency in the allocation of health resources [10]. We treat each of the community health center (CHC) under observation as a decision making unit. The basic idea is to compare the total output (*m*) obtained from each community health center (CHC) or DMUs designated as (*n*) after investing (*s*) amount of inputs or resources. Based on a systematic mathematical modeling, a combination of the output and input allows the construction of the production possibility frontier and decision making units (DMUs) or community health centers (CHC) located on the frontier can be said to be production efficient. Thus our C^2^R model of efficiency frontier for community health center (CHC) is expressed mathematically as:

1 $$ \left\{\begin{array}{l}M\mathrm{i} n\theta \\ {}\begin{array}{c}s.t.\sum \limits_{j=1}^n{X}_j{\lambda}_j\le \theta {X}_0\\ {}\sum \limits_{j=1}^n{Y}_j{\lambda}_j\ge {Y}_0{\lambda}_j\ge 0\end{array}\end{array}\right. $$

where *θ* is the efficiency variable, *λ*_*j*_ is the input and output weights of DMU_*j*_, and DMU_*j*_ is the valid value. Slack variables, *S*^−^ and *S*^+^, are the insufficient input and output parts which exist in the process of allocating health resources. When *θ* = 1, and *S*^+^ = 0, *S*^−^ = 0, then DMU_*j*_ is called DEA efficient. On the other hand, where *θ* = 1, but at least one input or output variable of the slack variables is greater than 0, then DMU_*j*_ is weakly DEA efficient. A DMU_*j*_ is non-DEA efficient at *θ* < 1. The ideal efficient value can be determined using:

2 $$ \left\{\frac{{X_i}^{\hbox{'}}=\theta {X}_i-{S}^i}{{Y_i}^{\hbox{'}}={Y}_i+{S}^{+}}\right. $$

where *S*^−^ is a slack of input variable and *S*^+^ is a slack variable of output variable.

Economic scale in the C^2^R model is based on:

3 $$ \sum \limits_{j=1}^n{\lambda}_j=k $$

where *k* is the economic scale of DMU. When *k* = 1, the economic scale is invariant; when *k* < 1, economic scale increases and the smaller the *k* value. This implies that increase in input will lead to a greater proportion of output. Moreover, when *k* > 1, the economic scale reduces and a high *k* value means a fast declining rate of efficiency of the DMU.

### Panel data model

The panel data model used in this study is constructed as follows

$$ {Y}_{it}={\alpha}_{it}+{\beta^{\hbox{'}}}_{it}{X}_{it}+{u}_{it} $$

$$ i=1,2,\dots, n;t=1,2,\dots, t $$

where *x*_*it*_ = (*x*_1*it*_, *x*_2*it*_…, *x*_*kit*_) as the vector from variables for the *k* dimension, *β*_*it*_ = (*β*_1*it*_, *β*_2*it*_…, *β*_*kit*_) as the corresponding explanatory variable vector *X*_*it*_ for 1 × *k* dimensional coefficient vector, *k* is the number of explanatory variables, *t* is the total number of each section of the observation period, *n* is the number of cross sections, and random error *u*_*it*_ is independent and satisfies the zero mean and equal variance. Based on the different constraints on the intercept and explanatory variable coefficients, the panel data model can be divided into three categories. To model the panel data, we used the number of diagnoses at CHC (ZLRC), number of health workers (RYS), number of beds (CWS), and public financial input allocated to CHC (CZTR) as explanatory variables. Using the total price value of investment in fixed assets in 1990 (100) as the base year, public financial investments are converted for CHC in 2008–2015. The Cobb-Douglas functional form of production functions was used to represent the relationship between outputs and inputs. This is mathematically expressed as:

$$ Y={AK}^{\alpha_1}{L}^{\alpha_2} $$

where *Y* is the total production, *A* is efficiency parameter, *K* is capital input, L is labor input, and *α*_1_,*α*_2_ is the output elasticity of capital and labor, respectively [16]. We used the Cobb-Douglas production function with the principle of analysis to convert the community health resource allocation efficiency of Jiangsu Province into output capacity of community health centers and explore the mathematical relationships between the input variables (human, material, financial resources) and the output. Thus, our modified Cobb-Douglas production function the diagnoses of community is the output (*Y*) while the number of health workers is the labor input, number of beds and public financial input in communities is the material input, and finance is the capital input. This is mathematically expressed as follows:

$$ Y={BX_1}^{\alpha_1}{X_2}^{\alpha_2} $$

On the other hand, we use logarithmic transformation to make linearization, and reduce the heteroscedasticity to obtain a linear panel data model function as follows:

$$ \ln Y=B+{\alpha}_1\ln {X}_1+{\alpha}_2\ln {X}_2 $$

Thus, our final panel data model based on Cobb-Douglas production function and general linear panel data model is mathematically expressed as:

4 $$ \ln {(ZLRC)}_{it}={\beta}_0+{\beta}_1\ln {(RYS)}_{it}+{\beta}_2\ln {(CWS)}_{it}+{\beta}_3\ln {(CZTR)}_{it}+{u}_{it} $$

where *ln*(*ZLRC*)_*it*_ is the output of diagnoses of community in 13 cities in Jiangsu Province; *i* is each city; *t* as the time or year; and *ln*(*RYS*)_*it*_, *ln*(*CWS*)_*it*_, *ln*(*CZTR*)_*it*_ are input of number of health workers, beds, and public financial input in communities in every city, every year of Jiangsu Province, respectively. *β*_1_, *β*_2_, *β*_3_ is the sensitive coefficient of diagnoses to health workers, beds, and public financial input in communities respectively, namely the efficiency coefficient. The greater the efficiency coefficient, the higher the allocation efficiency of community health resources is, and vice versa. *β*_0_ is the constant number as an evaluation parameter of overall efficiency; *u*_*it*_ is the disturbance as a random error.

To estimate the fixed effect model and random effects model, the Hausman Test was used and returned a score of *H* = 15.9027(*P* = 0.0012) which is an indication of the presence of fixed effect model. Moreover, *R*^2^ values of 0.68, 0.94, and 0.82 for the estimated pooled regression model, fixed-intercept model, and fixed-coefficient model suggest a significant model fit for the analysis. The covariance test was used to determine the specific form of the model and this returned an *F*_1_ test value of 7.33 > *F*_0.01_ (48,78), *P* < 0.01, hence, the selecting of fixed-intercept model while the *F*_2_ = 4.40 > *F*_0.01_(36,78), *P* < 0.01 means the selecting fixed-coefficient model. Table 1 shows the unit root test to examine the stability of the series as the panel data reflects two-dimensional information of time and cross section [11, 20]. Table 1 shows that ln(ZLRC) and ln(CZTR) in this study show an upward trend so we select intercept and trend item in unit root test. ln(ZLRC) and ln(CZTR) have no obvious tendency, so we select only intercept item in unit root test. According to common unit process test LLC and individual unit process ADF, the results of ln(ZLRC), ln(RYS), ln(CWS), and ln(CZTR) have passed significance test, *n* indicating that the variable does not have unit root; these variable series are all stationary time series (see Table 1).

**Table 1:** Community health center input by region from 2011 to 2015

	Southern zone	Middle zone	Northern zone
2011			
Number of doctors	108.64	91.46	65.12
	(124.92)	(75.04)	(76.70)
Number of nurses	114.03	86.61	76.13
	(142.98)	(99.34)	(101.97)
Number of pharmacists	23.82	26.03	18.11
	(27.19)	(22.72)	(17.74)
Number of other staff	45.45	41.66	35.03
	(51.28)	(40.49)	(42.77)
Number of beds	263.27	183.53	178.67
	(293.35)	(177.37)	(227.29)
2012			
Number of doctors	113.99	96.61	65.38
	(131.08)	(76.68)	(76.45)
Number of nurses	120.96	90.33	79.20
	(150.49)	(101.42)	(106.10)
Number of pharmacists	24.09	27.00	17.17
	(27.67)	(24.45)	(17.17)
Number of other staff	46.78	40.54	39.20
	(54.40)	(36.80)	(49.17)
Number of beds	272.43	189.57	183.41
	(298.99)	(185.16)	(231.38)
2013			
Number of doctors	138.92	96.86	67.40
	(149.89)	(80.03)	(80.51)
Number of nurses	156.49	94.44	84.45
	(194.47)	(103.13)	(122.39)
Number of pharmacists	28.14	26.49	18.32
	(30.18)	(23.02)	(17.98)
Number of other staff	55.43	39.91	40.83
	(64.54)	(33.78)	(54.16)
Number of beds	286.88	195.04	187.74
	(319.39)	(185.91)	(243.37)
2014			
Number of doctors	160.35	98.89	69.87
	(177.95)	(87.81)	(95.95)
Number of nurses	175.09	98.46	89.27
	(210.01)	(110.44)	(129.17)
Number of pharmacists	28.96	24.45	16.29
	(33.12)	(19.48)	(16.43)
Number of other staff	58.91	38.62	35.07
	(64.23)	(36.99)	(47.88)
Number of beds	306.42	199.72	201.26
	(336.77)	(188.48)	(260.82)
2015			
Number of doctors	156.78	100.44	71.13
	(163.49)	(84.33)	(94.52)
Number of nurses	184.52	90.88	109.08
	(216.58)	(107.79)	(219.54)
Number of pharmacists	30.12	24.30	16.30
	(33.52)	(20.71)	(16.88)
Number of other staff	63.66	41.66	38.52
	(74.82)	(41.29)	(59.24)
Number of beds	323.17	206.59	220.95
	(352.76)	(195.04)	(284.52)

### DEA model

We use DEAP 2.1 to conduct the regression of DEA model while Eviews 6.0 is used for the regression of the panel data model. Regression of the model from 2008 to 2015 can be obtained by the constant *β*_0_, the disturbance as random error (*u*_*it*_), and the sensitive coefficient (*β*_1,_*β*_2,_*β*_3_). The fixed-coefficient is presented in Table 2 where ZLRC is the number of diagnoses at CHC, RYS is number of beds, CWS is number of health workers, CZTR is the public financial input allocated to CHC, NJ as Nanjing, WX as Wuxi, CZ as Changzhou, NT as Nantong, LYG as Lianyungang, HA as Huaian, YC as Yangcheng, YZ as Yangzhou, ZJ as Zhenjiang, TZ as Taizhou, and SQ as Suqian.

**Table 2:** Fixed effect coefficients

Natural logarithm of variable	Coefficient
CONST.	66.716
RYS_NJ_	0.6397
CWS_NJ_	− 0.0937
CZTR_NJ_	0.5947
RYS_WX_	0.0789
RYS_WX_	0.2247
CWS_WX_	0.4234
RYS_XZ_	0.3222
RYS_XZ_	0.9501
CWS_XZ_	− 0.2323
RYS_CZ_	− 2.0111
RYS_CZ_	− 0.4341
CWS_CZ_	0.821
RYS_SZ_	0.1228
RYS_SZ_	0.1508
CWS_SZ_	0.1548
RYS_NT_	− 0.3341
RYS_NT_	− 0.3718
CWS_NT_	0.6795
RYS_LYG_	− 0.8376
RYS_LYG_	1.3207
CWS_LYG_	− 0.0885
RYS_HA_	0.0585
RYS_HA_	2.2226
CWS_HA_	− 0.37
RYS_YC_	0.1566
RYS_YC_	− 0.0232
CWS_YC_	0.339
RYS_YZ_	3.5743
RYS_YZ_	− 1.4431
CWS_YZ_	0.4503
RYS_ZJ_	0.0514
RYS_ZJ_	− 0.3428
CWS_ZJ_	0.2954
RYS_TZ_	− 0.2461
RYS_TZ_	1.2493
CWS_TZ_	0.2248
RYS_SQ_	− 1.4248
RYS_SQ_	− 0.3516
CWS_SQ_	− 0.0161

In the fixed-coefficient, *R*^2^ = 0.9801, *F* = 23.9078(*P* = 0.0000). In addition to the ln(RYS)_ZJ,_ ln(RYS)_WX_, ln(RYS)_CZ_, ln(CWS)_NJ_, ln(CWS)_SQ_, ln(CWS)_YC_, and ln(CZTR)_SQ_ do not pass the significance test; the rest pass the significance test, indicating that the model fits well.

## Results

### The evaluation of technical efficient and scale efficient (transverse dimension)

Table 3 shows the efficiency values and slack variables of community health resources of every city in Jiangsu Province in 2015 based on formula (1) of the C^2^R model. The table shows that the efficiency values of CHC in Xuzhou, Suzhou, Nantong, Yangzhou, and Lianyungang are relatively high. This is shown by the efficiency values of 1 in all cities and the slack variables of 0. This is an indication that on the production function curve, the existing conditions can achieve the maximum output or DEA technical efficiency [21, 22]. On the other hand, the efficiency values of the other cities are less than 1 and the corresponding slack variables are not all 0. This implies that the allocation efficiency of community health resources of the seven cities is non-DEA effective. This is especially in the case of Huaian and Taizhou where the efficiency values are only 0.59 (59%) and 0.51 (51%) respectively.

**Table 3:** Community health center output by region from 2011 to 2015

	Southern zone	Middle zone	Northern zone
2011			
Number of outpatients treated	415 443.00	130 649.35	97 829.24
	(443 102.45)	(129 884.62)	(127 678.84)
Number of inpatients treated	7 399.56	4 779.33	4 183.06
	(8 436.20)	(5 655.05)	(6 745.93)
2012			
Number of outpatients treated	453 061.69	135 897.25	102 745.80
	(482 991.17)	(134 747.30)	(137 877.10)
Number of inpatients treated	8 048.00	5 030.10	4 454.95
	(8 958.04)	(6 144.57)	(7 173.06)
2013			
Number of outpatients treated	491 763.43	133 940.04	109 064.17
	(524 629.77)	(132 661.98)	(147 501.68)
Number of inpatients treated	8 680.52	5 606.72	4 873.99
	(9 601.73)	(6 875.79)	(7 849.25)
2014			
Number of outpatients treated	603 252.83	148 146.02	116 661.30
	(1 049 100.16)	(140 858.75)	(161 280.77)
Number of inpatients treated	9 606.59	8 482.67	5 547.24
	(10 718.50)	(16 506.59)	(9 015.13)
2015			
Number of outpatients treated	676 363.45	151 725.69	123 562.72
	(1 470 044.55)	(146 355.18)	(175 891.95)
Number of inpatients treated	10 587.92	7 242.60	6 502.30
	(11 929.22)	(8 831.23)	(10 169.34)

On the other hand, Table 4 shows the actual and ideal values of community health resource input and output of non-DEA efficient cities in Jiangsu Province in 2015. The table shows that there is over-investment of health workers and infrastructure, in the economically developed areas in the South of Jiangsu. This means that health workers are drawn to the developed areas [23, 24]. For example, the number of excess health workers in Changzhou is most severe as the difference between the actual value and the ideal value is about 55 people. Further, the cost per visit to CHC is generally high in the Northern parts of the province and this is an indication of input redundancy. The difference between the actual value and the ideal value is about 36.10 Yuan in Huaian city alone. Again, the output deficiency in the daily number of inpatient stays, especially in Southern Jiangsu where manpower and material input is over-invested, shows that those resources are not fully and reasonably utilized. When the health investment of Zhenjiang and Nanjing are at the ideal value, the daily number of inpatient stays should also be increased by 1.01 and 0.85 days.

**Table 4:** Measures of efficiency by region from 2011 to 2015

	Southern zone	Middle zone	Northern zone
2011			
Overall efficiency	0.3549	0.2938	0.2026
Scale efficiency	0.6900	0.6354	0.4468
Pure technical efficiency	0.4991	0.4484	0.4400
2012			
Overall efficiency	0.3754	0.2710	0.1987
Scale efficiency	0.7162	0.6351	0.4352
Pure technical efficiency	0.5084	0.4140	0.4427
2013			
Overall efficiency	0.3897	0.3064	0.2223
Scale efficiency	0.7079	0.6579	0.4653
Pure technical efficiency	0.5340	0.4518	0.4636
2014			
Overall efficiency	0.2255	0.1762	0.1438
Scale efficiency	0.6847	0.5574	0.4063
Pure technical efficiency	0.3195	0.3066	0.3432
2015			
Overall efficiency	0.2259	0.1984	0.1587
Scale efficiency	0.6163	0.5772	0.3899
Pure technical efficiency	0.3555	0.3333	0.3948

Using formula (3), we calculated the economic scale of the input and output of the community health resources in Jiangsu Province. The results show an increase in the economic scale of five DMUs including Nanjing (1.10) and Zhenjiang (1.05) while that of two DMUs declined and this includes Taizhou (0.58) and Changzhou (0.73). Finally, the economic scales are invariant in five economic scale cities, indicating that inputs are neither too large nor too small at the best situation (Table 5).

**Table 5:** Malmquist Productivity Index and its decomposition by region, 2011 to 2015

	2004–2008	2004–2005	2005–2006	2006–2007	2007–2008
Southern zone					
Malmquist index	1.0413	1.0407	0.9505	1.0254	0.9753
Technological change	1.6361	0.9842	0.9154	1.7720	0.9736
Change in efficiency	0.6174	1.0258	1.0072	0.5612	0.9717
Change in scale efficiency	0.8666	1.0069	0.9588	0.9382	0.8732
Change in pure technical efficiency	0.6911	0.9881	1.0190	0.5803	1.0795
Sample size	256	256	256	256	256
Middle zone					
Malmquist index	1.1560	0.9762	0.9701	1.0835	1.0293
Technological change	1.7126	1.0583	0.8580	1.8847	0.9144
Change in efficiency	0.6548	0.8948	1.0968	0.5577	1.0919
Change in scale efficiency	0.8811	0.9693	1.0048	0.8218	1.0045
Change in pure technical efficiency	0.7209	0.8954	1.0588	0.6582	1.0543
Sample size	41	41	41	41	41
Northern zone					
Malmquist index	1.2793	1.0112	1.0194	1.1683	1.0042
Technological change	1.6337	1.0316	0.9107	1.8070	0.9098
Change in efficiency	0.7596	0.9508	1.0858	0.6271	1.0708
Change in scale efficiency	0.8465	0.9449	1.0369	0.8472	0.9307
Change in pure technical efficiency	0.8705	0.9760	1.0157	0.7181	1.1160

### Efficiency evaluation for allocation of community health resources

Table 5 on the other hand shows that health resource allocation efficiency in Jiangsu Province in 2008–2015 is relatively higher in south of Jiangsu cities such as Wuxi and Suzhou based panel data model. Overall efficiency of the economically underdeveloped regions such as Huaian and Suqian is low in the technical and scale efficient evaluation for Nanjing. Again, Table 5 shows that allocation efficiency of health workers is commonly not high. The under-development areas with relatively poor working environment could not attract amounts of health workers which may contribute to this. The working team is not stable and such medical education is not enough to meet the requirements of medical technique workers’ self-improvement and further study. The allocation efficiency of health workers is relatively low and cannot match the standard of community health centers. While the relatively economically developed area could attract mass health workers, it may even be over-investment from technical and scale efficient analysis even though allocation efficiency of health workers in these areas is not high. This may be because the working conditions of workforce are low and cannot match those from big hospitals hence the impact on working enthusiasm and service ability. We can conclude that the third-class hospitals have large proportion, it is over the standard and it is increasing continuously. The more patients prefer big hospitals so the amount of patients seeking medical advice and the in-hospital ratio in the surrounding community health centers drop sharply to waste the community health resources. From the aspect of financial involvement efficiency, the developed cities get more population, better community health centers are set up, and the financial involvement efficiency is higher. On the contrary, the efficiency is lower.

### The vertical dimension

In Table 5, it is observed that in 2008 and 2015 the efficiency value was 1 and the entire slack is 0; the allocation efficiency is relatively high in these years. From 2009 to 2014, the technical efficiency is less than 1 and the allocation efficiency is non-effective. In 2008 and 2015, allocation efficiency of Jiangsu Province is in a state of effective and economic scale is invariant. From 2009 to 2014, the economic scale is less than 1 and the tendency of economic scale is increased. Especially in 2009 and 2011, technical and scale efficient values are below 0.6; in 2012, the efficiency value basically returned to effective. From the perspective of input, Jiangsu Province proposed the urban standard of CHC after 2008, so bed resources showed the phenomenon of redundant input from 2009 to 2012; after the Deepen Medical Reform in 2012, health worker resources showed redundant input; from the perspective of output, the community health resource output was insufficient since 2009, but with the Deepen Medical Reform, public decision makers adjusted the non-effective condition of community health resource allocation and the phenomenon of insufficient output had improved (Table 5).

## Discussion

### Lateral comparison of health resource allocation among CHC

Our study sought to evaluate the efficiency in the allocation of resources to community health centers (CHCs) in selected cities in Jiangsu Province based on an evaluation of longitudinal data. From the analysis, a number of conclusions can be made as follows:

Firstly, there is evidence of high investment and low returns of Community Health resources in many parts of the province. Overall, the study showed that in 2015, more than 50% of health resources allocated to CHCs under the studied cities in Jiangsu Province were not used efficiently. Two cities in economically less-developed northern parts of the province (see Table 3) proved to be highly DEA inefficient: A case that supports the argument that more health resources are wasted among the economically less-developed areas of the province. We found out that this serious DEA inefficiency is largely a result of the high cost of visits to CHC without corresponding social values which ultimately affects the basic health service requirements of patients. Another reason is that some districts do not follow the regulations of the regional health program intended to reduce waste of health service resources. From Table 4, it can be observed that the daily number of inpatient stay in the southern cities in Jiangsu where more health workers and material resources are invested is not high leading to underutilization of health resources while high demand northern areas have fewer resources. Thus, realigning resource distribution is an essential step towards alleviating this resource lag. Specifically, a new mechanism that will ensure the rapid transfer of resources among CHC or cities to eliminate wastes and over demand is imperative.

Secondly, there is evidence of improved efficiency in the allocation of community health resources but greater variation still exists. Generally, it is obvious that Southern cities and municipalities are more proactive in terms of health resource efficiency while northern areas of the province still lag behind. For example, Zhenjiang Municipality initiated “3+X” family health responsibility team to make use of health human resource as much as possible and to improve human resource allocation efficiency. To improve the bed resource allocation efficiency, Zhenjiang implemented an integrated management and transfer treatment system between community health service centers and group hospitals to ensure shared resource. While XuZhou city formulated and implemented a new basic medical health center compensation mechanism, YanCheng enhanced the financial allowance and incorporated it into budget management to improve the financial resource allocation efficiency. Thus, there is the need for less-efficient communities to learn from successful once to ensure even efficiency in public health expenditure security mechanism and enhance the construction of community health service centers standardization.

Thirdly, there is the need to improve the economic scale of community health resources. If economic scale decreases progressively between two cities, there is the need to interrogate the causative factors and not simply reduce health resource input blindly. It is essential to learn from cities with superior economic scale to help lower ones to increase input properly and optimize resource allocation. This is because it is beneficial for the health operation department to adjust local health resource allocation and disposition in time to optimize health resource allocation and improve health resource utilization efficiency, finally, which is consistent with local health programmer by analyzing economic scale.

### Longitudinal comparison of health resource allocation among cities

From a longitudinal comparison of health resource allocation, it is important to note that before the Deepen Medical Reform, public decision makers had not paid enough attention to investment of community health resources in Jiangsu; hence, technical and scale efficiency value of resources remained low from 2009 to 2011. After the Deepen Medical Reform, Jiangsu Province has strengthened the standardization of the construction of community health service, explored the community health development model to suit local conditions, promote the equalization of basic public service and rational allocation and utilization of health resources, and guarantee the basic medical care; through these measures, allocation efficiency of community health resources generally increased in Jiangsu Province.

Secondly, there is lack of effective long-term and sustainable community health resource allocation mechanism. Although there is some modicum of technical efficiency, the analyzed data suggest that there are irregular changes in return to scale which signifies an inability to maintain effectiveness for a long time to ensure overall efficiency in health resource allocation in Jiangsu Province.

## Conclusion

The study has some implications for Jiangsu Province and China as a whole. Ying [23] noted widespread inefficiencies of healthcare resource utilization in other provinces in China. Zhang [24] also contends that several countries (Sweden, UK, etc.) face similar challenges of technical and scale inefficiency, resource redundancy, under-resourced healthcare facilities, etc. The major difference in the case of China and other countries is the variation in positioning value orientation of health resource allocation, role of market forces, and the governmental functional orientation to resource allocation [24].

The provincial government has a number of options to correct the anomalies indicated in this study but are not accounted for in the new Healthy China 2020 Agenda. Firstly, Jiangsu Province must adopt a new model to allocate health resource and service utilization. This model must seek to eliminate redundancy in places where resources are not needed. Prior research studies have proposed the shifting of healthcare professionals (clinical and non-clinical) from areas with excess availability of human resources to underserved areas such as the northern parts of Jiangsu Province. One main approach is to provide incentive packages for health professionals to attract them to less-developed areas, but this has not eliminated the problem. An alternative is to revamp the healthcare supply chain system to ensure quick transfer of healthcare resources to backward areas. The rapid expansion of transportation system in China can be a strong partner in this respect. This can reduce the tendency to force professionals to stay in areas they least prefer. This can ultimately lead to an integrated regional health planning that facilitates inter-facility cooperation between medical centers to actively share medical resources.

Jiangsu Province must cultivate talents. There is the need to build the capacities of rural health human resources by instituting training activities in less-developed regions. For example, Guangxi Province has initiated a policy to provide support for medical and healthcare trainee. These students are then made to sign a bond to remain in service to the community after their training over a period of time. This approach has the potential to guarantee the availability of a recognizable number of healthcare professionals and quality healthcare over a period of time to reduce inequity in access to quality healthcare.

Secondly, the province must educate citizens on the need to utilize community health service centers for primary healthcare purposes. There should be a clear mandatory policy for ailing residents to first seek services of community health centers while seriously ill residents attend the hospitals. This can boost the net utilization of services to avoid redundancy. Similarly, there is the need to ensure favorable compensation ratio of health insurance for community health centers and institutes to guarantee their survival and ability to offer the same quality healthcare service they set up to provide. This should be done in tandem with strengthening the establishment of other grass root medical institutions that will help to reduce the observed redundancies in inputs and overall service utilization. Thirdly, there is the need to reshape the structure of healthcare resources and its allocation. Optimizing the input/output and controlling the scale of operations could improve the efficiency of resource allocation and service utilization in all the provinces yet this is not possible without the presence of healthcare facility managers with strong management skills. Like most healthcare facilities in China, doctors with little training in administration are in charge of the day to day operations of the healthcare facilities. It is advisable to appoint highly qualified healthcare administrators to work in the sector. The province must provide training programs for public health, rural health, and urban health management professionals and technical personnel to support their management of healthcare facilities in line with modern approaches.

Future research can focus on designing questionnaires and selecting typical indexes making sure the evaluation meets the aims. We can also divide the sample in a further step, evaluating the health resources in cities and the rural areas, which can make the results be more convincing.

## Abbreviations

CHC: Community health center
DEA: Data envelopment analysis
DMUs: Decision making units
